# Concise Analysis of Single-Stranded DNA of Recombinant Adeno-Associated Virus By Automated Electrophoresis System

**DOI:** 10.1089/hum.2023.148

**Published:** 2024-02-15

**Authors:** Yuzhe Yuan, Kiyoko Higashiyama, Noriko Hashiba, Kyoko Masumi-Koizumi, Keisuke Yusa, Kazuhisa Uchida

**Affiliations:** Graduate School of Science, Technology and Innovation, Kobe University, Chuo-ku, Kobe, Japan.

**Keywords:** recombinant adeno-associated virus, inverted terminal repeat, single-stranded DNA, automated electrophoresis

## Abstract

Recombinant adeno-associated virus (rAAV) is a prominent viral vector currently available for human gene therapy. The diameter of the rAAV capsid is ∼25 nm, and a positive or negative single-stranded DNA is packaged within the vector capsid. In this report, we describe a concise method to examine the extracted rAAV genome using an automated electrophoresis system. The rAAV genome, prepared from vector particles through either heat treatment at 95°C for 10 min or the phenol–chloroform extraction method, was analyzed using an automated electrophoresis system under denaturation conditions. The heat treatment protocol demonstrated a comparable yield with the phenol–chloroform extraction protocol, and the quantified amounts of the rAAV genome obtained using the automated electrophoresis system were consistent with those quantitated by quantitative PCR. Additionally, crude rAAV extractions could also be analyzed by the automated electrophoresis system after DNase I treatment. These results indicated that this simple and quick analysis using automated electrophoresis is highly useful for confirming the purity and integrity of the rAAV genome.

## INTRODUCTION

Recombinant adeno-associated virus (rAAV) has been widely used in gene therapy as a vector to transfer genes to patients with genetic disorders.^1,2^ rAAV has several advantages, including its ability to infect dividing and nondividing cells and its low potential for causing an inflammatory response, although AAV2 has recently been reported as a suspicious etiological factor causing severe acute hepatitis in children.^3–5^ The wild-type AAV genome is a single-stranded DNA (ssDNA) of ∼4.7 kb encoding Rep, Cap, membrane-associated accessory protein,^6^ and assembly-activating protein^7^ enclosed within two, 145-base, inverted terminal repeats (ITRs).^8,9^

rAAV is constructed by replacing viral genes with the gene of interest (GOI) to be expressed in target cells, thus the packaging capacity is limited to ∼5 kb.^1,2,10,11^ The clinical dose of rAAV is determined based on the vector genome (vg) titer per mL and it requires the availability of accurate quality control methods. Quantitative real-time PCR (qPCR) and droplet digital PCR (ddPCR) are the primary analytical methods widely used in quantification of rAAV owing to their robustness and wide measurement range.^12–19^ Transgene encapsidation is not highly efficient in the vector manufacturing process, and the produced rAAV still contains empty and partially loaded components, which are considered to be impurities without therapeutic function.^20,21^ Analytical ultracentrifugation (AUC)^22–24^ and transmission electron microscopy (TEM)^25,26^ are currently used for analysis of the products. AUC is used to fractionate empty and filled rAAV derivatives and can also be used to determine the relative proportion of partially filled capsids in an AAV batch,^27^ and TEM could morphologically discriminate empty particles from filled particles.^28,29^ Mass photometry has also been reported as a method to characterize capsids based on their mass, distinguishing empty, partially filled, full, and overloaded capsids.^30–32^ Traditionally, verification of the rAAV genome size has been conducted through denaturing agarose gel electrophoresis.^33,34^ These methods are time-consuming and have limited precision owing to the need for optimization of the analytical conditions. Recently, alternative approaches such as capillary electrophoresis (CE)^34,35^ or electrophoresis-mediated microfluidics^36^ have been reported.

The aim of our study was to examine whether the TapeStation system can be used for the analysis of rAAV ssDNA under denaturation conditions. It is frequently employed for sample quality control of both DNA and RNA for massive parallel sequencing. RNA was concisely analyzed under denaturation conditions using the RNA ScreenTape^®^ to analyze size, quantity, and integrity. In this study, we show that the analysis of the single-stranded form of the rAAV genome using the TapeStation system is fast, concise, and relatively robust for examining the purity, quantity, and integrity of the rAAV genome.

## MATERIALS AND METHODS

### Plasmid, DNA, and rAAV

The following reagents were used for automated electrophoresis: pAAV-ZsGreen1 (Takara Bio Inc., Kusatsu, Japan), a 1-kb DNA ladder (New England Biolabs Ltd., Ipswich, MA, USA), and an RNA ladder (Agilent Technologies, Inc., Santa Clara, CA, USA). To prepare the rAAV-ZsGreen1 DNA, 10 μg of the plasmid was digested with PvuII (New England Biolabs) and *Dra*I (New England Biolabs), purified by phenol–chloroform extraction, and precipitated with ethanol.

Purified AAV8-AAT-FIXp and the crude extracts of rAAV2-CMV-ZsGreen1 prepared from VPC2.0 cells on days 1, 2, and 3 post-transfection were provided by the Manufacturing Technology Association of Biologics. The rAAV samples, 1908_rAAV1-CMV-ZsGreen1, 1909_rAAV2-CMV-ZsGreen1, and 1911_rAAV6-CMV-ZsGreen1, were also provided by the Manufacturing Technology Association of Biologics. For rAAV production, 293T cells (American Type Culture Collection, Manassas, VA, USA) were cultured in Dulbecco's modified Eagle's medium (DMEM) (Sigma-Aldrich Co., St. Louis, MO, USA) supplemented with 10% fetal bovine serum (Biowest, Bradenton, FL, USA). PEIpro (Polyplus Transfection, Vandœuvre-lès-Nancy, USA) was used for transfection with pAAV-ZsGreen1, pHelper (Takara Bio Inc.), and rep-cap plasmids (pRC1, pRC2-mi342, or pRC6; Takara Bio Inc.) in 293T cells. The ratio of PEIpro (Polyplus Transfection) to DNA weight was 1:1 in serum-free DMEM. After 72 h post-transfection, cells were lysed with Triton X-100 buffer (0.5% Triton X-100 and 2 mM MgCl_2_ in phosphate-buffered saline [PBS]). The volume of the cell lysate was reduced to 1/8 using tangential flow filtration (Spectrum Inc., Rancho Dominguez, CA, USA) and applied to a HiTrap AVB Sepharose Column (GE Healthcare, Chicago, IL, USA). After washing, rAAV was eluted with 50 mM glycine-HCl (pH 2.7). Further purification of rAAV was performed by CsCl density gradient centrifugation at 148,500 *g* for 46 h at 21°C. Full rAAV particles were then subjected to dialysis against PBS and stored at −80°C until use.

### Electrophoresis of DNA in the denaturation condition

The 4150 TapeStation System (Agilent Technologies, Inc.) was used for DNA sample electrophoresis. The system uses microfluidic technology to perform CE: nucleic acid molecules migrate through a gel matrix within the tape in an electric field, and the rate of migration is determined by the size and charge of the molecules. For DNA loading, an equal volume of the loading buffer (high-sensitivity [HS] RNA loading buffer) was added, and the mixture was incubated at 75°C for 5 min. Electrophoresis was performed using an HS RNA ScreenTape (Agilent Technologies, Inc.). The quantitative range was 0.5–10 ng/μL and sizing range was 0.1–6.0 kb.

### rAAV genome DNA extraction from rAAV samples

For analysis of rAAV genomic DNA extracted from rAAV particles (1908_rAAV1-CMV-ZsGreen1, 1909_rAAV2-CMV-ZsGreen1, and 1911_rAAV6-CMV-ZsGreen1), 1.5 × 10^9^ rAAV capsids (20 μL) were destroyed by phenol–chloroform extraction, and the extracted genomic DNA was purified using AMPure XP beads (Beckman Coulter Inc., Brea, CA, USA). Phenol–chloroform extraction was performed twice with an equal volume of phenol: chloroform: isoamyl alcohol (25:24:1), followed by chloroform extraction twice. To 20 μL of genomic DNA (2.9 ng/μL), 36 μL of AMPure XP beads was added, and the genomic DNA was washed twice with 50 μL of 70% ethanol by magnetic precipitation and eluted with 10 μL of 10 mM Tris-HCl and 1 mM EDTA (pH 8.0).

Three rAAV genomic DNA extraction protocols were performed using 1908_rAAV1-CMV-ZsGreen1: protocol #1, heat treatment of purified rAAV capsids at 95°C for 10 min; protocol #2, phenol–chloroform extraction/purification with AMPure XP beads (Beckman Coulter Inc.), as described above; and protocol #3, heat treatment (95°C for 10 min) and purification with AMPure XP beads.

### qPCR and ddPCR

All primers and probes were custom synthesized and high-performance liquid chromatography purified (Eurofins Genomics K.K., Tokyo, Japan). For rAAV-ZsGreen1 DNA, the forward primer 5′-TTCGTGATCACCGGCGAGGGCAT-3′, reverse primer 5′-CCGTACATGAAGGCGGCGGACAA-3′, and probe [FAM]AACCTGTGCGTGGTGGAGGGCGGC[BHQ1] were used for qPCR and ddPCR. For qPCR, pAAV-ZsGreen1 DNA (Takara Bio Inc.) digested with PvuII (New England Biolabs) was used to generate a standard curve. The reaction mixture comprised primers and probes at a final concentration of 0.4 and 0.1 μM for each using the QuantiTect Probe PCR Kit (204343; Qiagen, Hilden, Germany) in a final volume of 20 μL. qPCR was performed using the StepOnePlus system (Applied Biosystems, Waltham, MA, USA). The cycling conditions were as follows: 15 min at 95°C, followed by 40 cycles of a two-step thermal profile comprising 15 s at 94°C and 60 s at 60°C. Each DNA template was tested in triplicate at two dilutions.

For ddPCR, the reaction mixture comprised 10 μL of ddPCR Supermix for probes (Bio-Rad Laboratories, Hercules, CA, USA), 2 μL of primers (final concentration of each primer was 0.9 μM), 1 μL of probe (final concentration 0.25 μM), and 1 μL of the template diluted with TE buffer (10 mM tris(hydroxymethyl)aminomethane-HCl, pH 8.0, 1 mM ethylenediaminetetraacetic acid) containing 0.01% of PF-68 in a final volume of 20 μL. Each DNA template was tested in triplicate at two dilutions. The plates were transferred to a QX200 Automated Droplet Generator (Bio-Rad Laboratories). A 96-well plate containing the generated droplets was transferred to a C1000 Touch Thermal Cycler (Bio-Rad Laboratories). The cycling conditions were as follows: 10 min at 95°C, followed by 40 cycles of a two-step thermal profile comprising 30 s at 94°C and 60 s at 60°C. The plate was then transferred to a QX200 droplet reader (Bio-Rad Laboratories), and data analysis was performed using QuantaSoft software (version 1.7.4.0917; Bio-Rad Laboratories). The threshold separating the negative and positive droplets was manually set just above the cluster of negative droplets or just below the cluster of positive droplets, respectively.

## RESULTS

### Denaturation of double-stranded DNA into ssDNA

**Figure 1.**
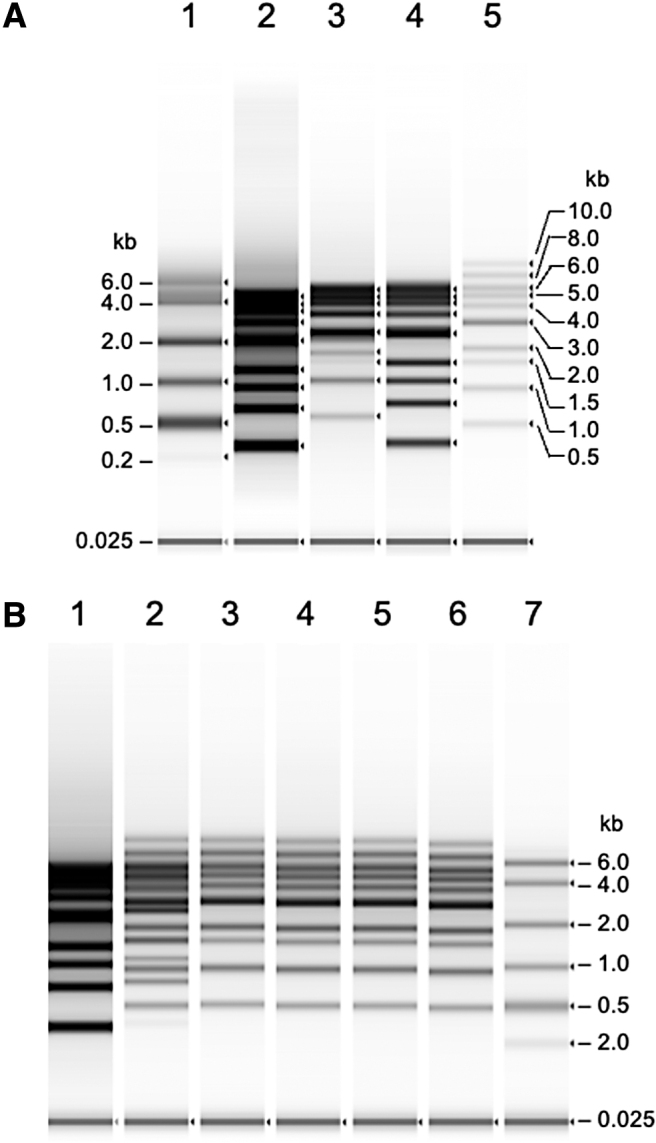
Analysis of DNA ladder by TapeStation with an HS RNA ScreenTape. **(A)** Analysis of a 1-kb DNA ladder with and without heat treatment. A 0.5 volume of HS RNA sample buffer was added to the HS RNA ladder, and the mixture was loaded after heat treatment at 72°C for 3 min, as per the manufacturer's protocol (lane 1). A 0.5 volume of HS RNA sample buffer was added to a 1-kb DNA ladder (12.5 ng/μL), and the mixture was loaded without heat treatment (lane 2). A 0.5 volume of HS RNA sample buffer was added to a 1-kb DNA ladder (12.5 ng/μL), and the mixture was loaded after heat treatment at 72°C for 3 min (lane 3). An equal volume of HS RNA sample buffer was added to a 1-kb DNA ladder (12.5 ng/μL), and the mixture was loaded without heat treatment (lane 4). An equal volume of HS RNA sample buffer was added to a 1-kb DNA ladder (12.5 ng/μL), and the mixture was loaded after heat treatment at 75°C for 5 min (lane 5). **(B)** An equal volume of HS RNA sample buffer was added to a 1-kb DNA ladder (12.5 ng/μL), and the mixture was loaded with heat treatment at 75°C for 0 min (lane 1), 3 min (lane 2), 5 min (lane 3), 7 min (lane 4), 10 min (lane 5), and 15 min (lane 6) using the HS RNA ScreenTape. Denaturation of a 1-kb dsDNA ladder after addition of HS RNA sample buffer; 0.5 volume of HS RNA sample buffer was added to the HS RNA ladder and the mixture was loaded after heat treatment at 72°C for 3 min, as per the manufacturer's protocol (lane 7). HS, high-sensitivity.

We first established the analytical conditions for ssDNA with a 1-kb DNA ladder containing 10 DNA fragments (0.5, 1.5, 1.0, 2.0, 3.0, 5.0, 6.0, 7.0, 8.0, and 10.0 kbp) using the TapeStation system. We analyzed double-stranded DNA (dsDNA) fragments using RNA ScreenTape for ssDNA analysis (Fig. 1). According to the manufacturer's instructions for RNA denaturation, we added a 0.5 volume of RNA sample buffer to the RNA sample and subjected the mixture to heat treatment at 72°C for 3 min. This denaturation condition was sufficient to linearize the RNA ladder (Fig. 1A, lane 1), but did not provide clear resolution for dsDNA with and without heat treatment (Fig. 1A, lanes 2 and 3). These results indicated that addition of a 0.5 volume of RNA sample buffer to the dsDNA mixture (1-kb ladder) was not sufficient for denaturation of dsDNA into a single-stranded form, even with heat treatment suitable for RNA denaturation. Next, we added an equal volume of RNA sample buffer to the 1-kb DNA ladder and compared the electropherograms with and without heat treatment at 75°C for 5 min (Fig. 1A, lanes 4 and 5). It should be noted that addition of an equal volume of the denaturation solution, along with heat treatment, provided a clear resolution of the 10 ssDNA fragments, and the mobility and fluorescence intensity of the DNA ladder changed compared with the bands without heat treatment (Fig. 1A). These results indicated that the dsDNA fragments were successfully denatured into a single-stranded form. The decrease in fluorescence intensity of ssDNA indicates the loss of intercalated fluorescent molecules between the complementary strands. In addition, the mobility of ssDNA fragments was consistent with the size of RNA (Fig. 1B). Taken together, RNA ScreenTape can also be used for dsDNA, although the denaturation condition for RNA (addition of 0.5 volume of HS RNA sample buffer with heat treatment at 73°C for 3 min) instructed by the manufacturer was not sufficient for complete denaturation of dsDNA (Fig. 1, lanes 3 and 5). For complete denaturation of dsDNA, addition of an equal volume of the sample buffer with incubation at 75°C for 5 min was required, and the denaturation conditions were valid for dsDNA up to 10 kb in length. Longer heat treatment, until 15 min, did not improve dsDNA denaturation (Fig. 1B).

### Denaturation of rAAV dsDNA and ITRs

**Figure 2.**
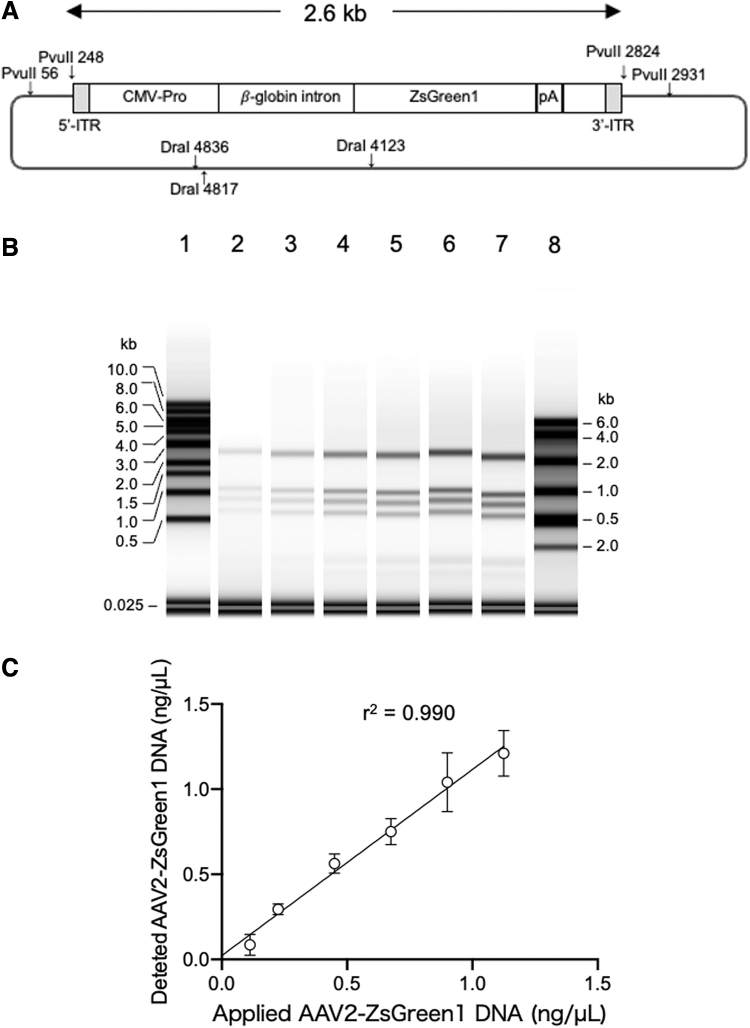
Quantification of 2.6-kb rAAV-ZsGreen1 DNA fragment generated from pAAV-ZsGreen1. **(A)** The structure of pAAV-ZsGreen1 and its digestion sites for PvuII and *Dra*I. **(B)** An equal volume of HS RNA sample buffer was added to a 1-kb DNA ladder (10 ng/μL), and the mixture was loaded after heat treatment at 72°C for 5 min (lane 1). An equal volume of HS RNA sample buffer was added to PvuII/*Dra*I-digested pAAV-ZsGreen1 at the following concentrations: 0.11 ng/μL (lane 2), 0.23 ng/μL (lane 3), 0.45 ng/μL (lane 4), 0.68 ng/μL (lane 5), 0.90 ng/μL (lane 6), and 1.10 ng/μL (lane 7); 0.5 volume of HS RNA sample buffer was added to the HS RNA ladder, and the mixture was loaded after heat treatment at 72°C for 3 min (lane 8). **(C)** Detected concentration of the 2.6-kb rAAV-ZsGreen1 fragment by TapeStation using an RNA screen strip was plotted against the loaded concentration determined by Qubit. The RNA sample buffer contained a 25-base standard oligonucleotide (0.7 ng/μL) for quantification. AAV, adeno-associated virus; ITR, inverted terminal repeat.

Next, we confirmed the denaturation of the rAAV-ZsGreen1 dsDNA fragment generated from pAAV-ZsGreen1 digested with PvuII and *Dra*I. As shown in Fig. 1, an equal volume of RNA sample buffer was added to the digested DNA fragments, and the mixture was incubated at 75°C for 5 min. The DNA mixture contained a 2,577-bp rAAV-ZsGreen1 fragment as well as 1,194-, 935-, 692-, 192-, 107-, and 19-bp fragments from the pUC portion (Fig. 2A). The electropherogram revealed that the mobility of rAAV-ZsGreen1 corresponded to a length of 2.6 kb based on the denatured 1-kb DNA ladder and RNA ladder (Fig. 2B). Quantification of rAAV-ZsGreen1 ssDNA using RNA ScreenTape was consistent with the amount of DNA applied after denaturation (Fig. 2C). These results indicate that the autoelectrophoresis system can be used to quantify rAAV ssDNA.

**Figure 3.**
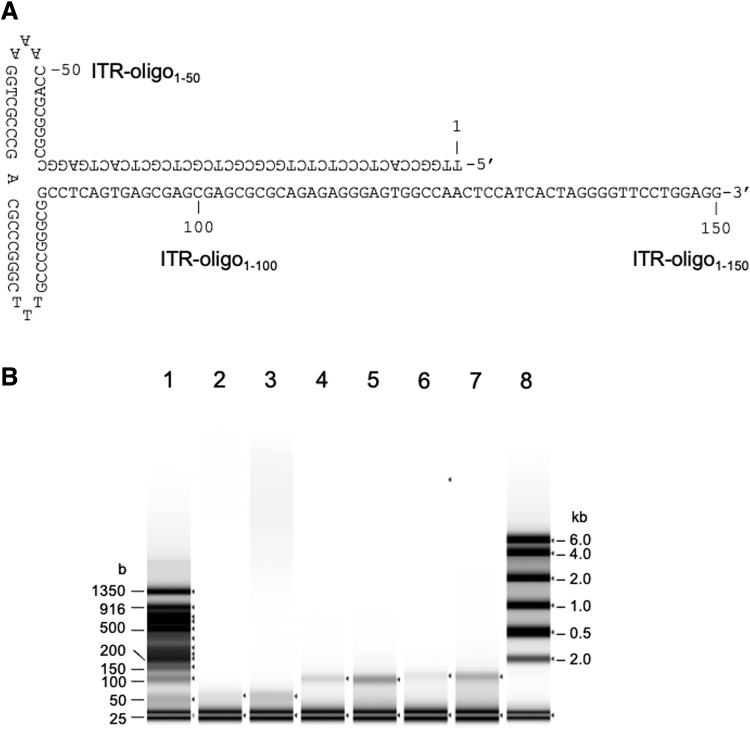
Analysis of synthesized ITR oligonucleotide. **(A)** Sequences of the synthesized ITR-oligo_1–50_, ITR-oligo_1–100_, and ITR-oligo_1–150_. **(B)** ITR-oligos were analyzed after addition of an equal amount of HS RNA sample buffer into oligo (10 ng/μL) with or without heat treatment at 75°C for 5 min. ITR-oligo_1–50_ without (lane 2) and with heat treatment (lane 3). ITR-oligo_1–100_ without (lane 4) and with (lane 5) heat. ITR-oligo_1–150_ without (lane 6) and with (lane 7) heat. An equal volume of HS RNA sample buffer was added to a 50-kb DNA ladder (10 ng/μL) with heat treatment at 75°C for 5 min (lane 1); 0.5 volume of HS RNA sample buffer was added to the HS RNA ladder, and the mixture was heated at 72°C for 3 min (lane 8).

The rAAV genome contained a GC-rich and palindromic ITR at both ends, which formed a T-shaped secondary structure (Fig. 3A). To address whether the denaturation conditions for dsDNA were also effective in linearizing the ITR secondary structure, we analyzed three synthesized oligonucleotides, ITR-oligo_1–50_, ITR-oligo_1–100_, and ITR-oligo_1–150_, using the TapeStation system after addition of an equal volume of RNA sample buffer with or without heat treatment at 75°C for 5 min (Fig. 3B). Without heat treatment, the intensities of the 50-, 100-, and 150-base bands were faint, but became clearer after heat treatment, indicating that partial linearization of the ITR-oligos occurred without treatment and was successfully completed after heat treatment. The oligonucleotides exhibited size-dependent mobility compared with that of the ssDNA ladder. These results confirmed that the denaturation conditions were also effective for ITR structures.

### Analysis of rAAV DNA extracted by phenol–chloroform

**Figure 4.**
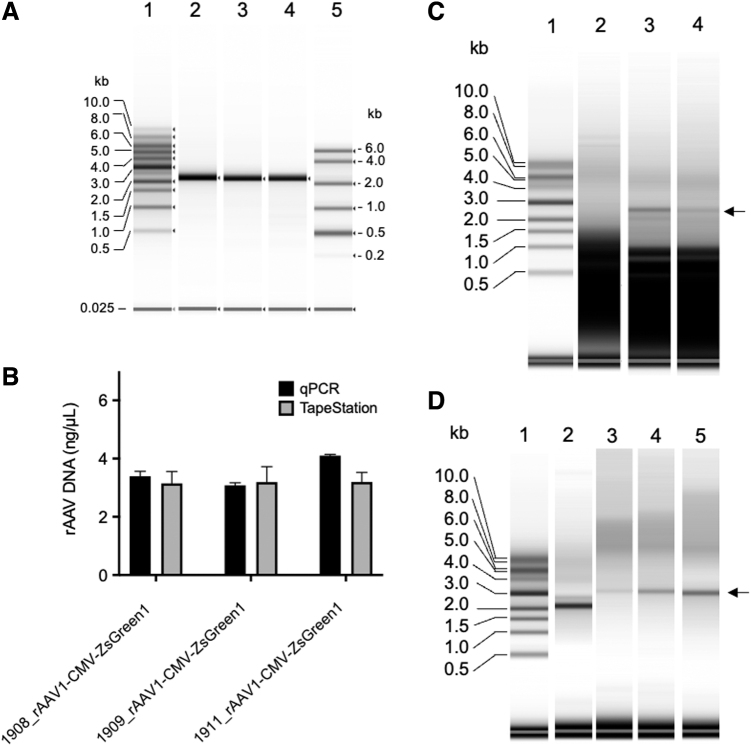
Analysis of rAAV genome pretreated using the phenol–chloroform extraction method. **(A)** rAAV DNA was prepared from 1908_rAAV1-CMV-ZsGreen1, 1909_rAAV2-CMV-ZsGreen1, or 1911_rAAV6-CMV-ZsGreen1 by the phenol–chloroform extraction method, and the extracted rAAV DNA (10 ng/μL) was mixed with an equal volume of HS RNA sample buffer and treated at 75°C for 5 min. The mixtures were analyzed by TapeStation using HS RNA ScreenTape. Lane 1, 1-kb DNA ladder (10 ng/μL); lane 2, 1908_rAAV1-CMV-ZsGreen1; lane 3, 1909_rAAV2-CMV-ZsGreen1; lane 4, 1911_rAAV6-CMV-ZsGreen1; and lane 5, 0.5 volume of HS RNA sample buffer was added to the HS RNA ladder and the mixture was heated at 72°C for 3 min. **(B)** Concentration of 1908_rAAV1-CMV-ZsGreen1, 1909_rAAV2-CMV-ZsGreen1, and 1911_rAAV6-CMV-ZsGreen1 determined by qPCR and TapeStation using the HS RNA ScreenTape. **(C)** rAAV2-CMV-ZsGreen1 extracted from the CVP2.0 cells on days 1, 2, and 3 post-transfection was treated with DNase I. The rAAV2-CMV-ZsGreen1 DNA sample was analyzed by TapeStation using the HS RNA ScreenTape. Lane 1, 1-kb DNA ladder (10 ng/μL); lane 2, day 1 sample (0.5 × 10^7^ vg/μL); lane 3, day 2 sample (1 × 10^8^ vg/μL); and lane 4, day 3 sample (0.5 × 10^8^ vg/μL). The *arrow* indicates 2.6-kb rAAV2-CMV-ZsGreen1. **(D)** rAAV8-AAT-FIXp (1 × 10^8^ vg/μL) DNA was prepared by the phenol–chloroform extraction method, and the extracted rAAV DNA (10 ng/μL) was mixed with an equal volume of HS RNA sample buffer and treated at 75°C for 5 min. The mixtures were analyzed by TapeStation using the HS RNA ScreenTape. Lane 1, 1-kb DNA ladder (10 ng/μL); lane 2, 3 ng/μL 1908_rAAV1-CMV-ZsGreen1; and lane 3, 0.17 ng/μL, lane 4, 0.67 ng/μL, and lane 5, 1.7 ng/μL AAV8-AAT-FIXp. The *arrow* indicates 3.2-kb AAV8-AAT-FIXp DNA. qPCR, quantitative real-time PCR; rAAV, recombinant adeno-associated virus.

To apply this assay to rAAV, we prepared ssDNA from three purified rAAV samples, 1908_rAAV1-CMV-ZsGreen1, 1909_rAAV2-CMV-ZsGreen1, and 1911_rAAV6-CMV-ZsGreen1, using the phenol–chloroform extraction method. The extracted ssDNA was analyzed using a TapeStation system with RNA ScreenTape after the denaturation treatment described above (Fig. 4A, lanes 2, 3, and 4). The intact rAAV genome packaged in the capsids was 2.6 kb in size. The extracted rAAV DNA revealed clear resolution without other bands, indicating the absence of a truncated partial genome and DNA impurities. The concentrations of rAAV ssDNA were 3.15 ng/μL for 1908_rAAV1-CMV-ZsGreen1, 3.19 ng/μL for 1909_rAAV2-CMV-ZsGreen1, and 3.19 ng/μL for 1911_rAAV6-CMV-ZsGreen1. The same samples were also quantitated by qPCR, and the concentration of rAAV was 3.09 ng/μL for 1908_rAAV1-CMV-ZsGreen1, 3.08 ng/μL for 1909_rAAV2-CMV-ZsGreen1, and 4.10 ng/μL for 1911_rAAV6-CMV-ZsGreen1 (Fig. 4B). The concentration of rAAV DNA quantified by the TapeStation system was almost consistent with that determined by qPCR, although the effective concentration range of ssDNA measurements was not as broad as that of qPCR.

The crude extracts of rAAV2-CMV-ZsGreen1 prepared from VPC2.0 cells transfected with the triple plasmids on days 1, 2, and 3 (Fig. 4C, lanes 2, 3, and 4) post-transfection were analyzed by TapeStation following DNase I treatment. rAAV2-CMV-ZsGreen1 DNA (2.6 kb) could not be detected on day 1, but the 2.6-kb viral genome in the crude extracts could be detected on days 2 and 3. These results indicated that this analytical system was also useful to monitor rAAV genome DNA containing DNA impurities. Factor IX-associated hemophilia, known as hemophilia B or Christmas disease, results from a deficiency or dysfunction of clotting factor IX. Sustained therapeutic factor IX levels were attained after liver-directed gene therapy with AAV vectors in patients with hemophilia B.^37,38^ We analyzed rAAV DNA carrying the FIX gene (AAV8-AAT-FIXp) as a GOI encapsulated in an AAV8 capsid (Fig. 4D).

### Analysis of rAAV DNA extracted by heat treatment

**Figure 5.**
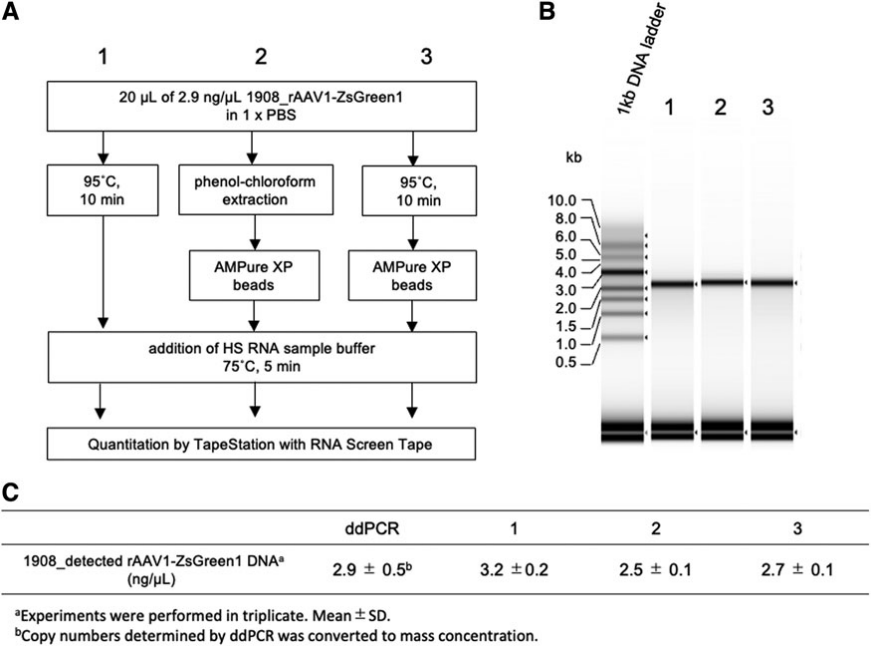
rAAV DNA preparation and measurement on RNA ScreenTape. **(A)** Three DNA extraction methods for 1908_rAAV1-CMV-ZsGreen1: 1, heat treatment; 2, phenol–chloroform extraction/AMPure XP purification; and 3, heat treatment/AMPure XP purification. **(B)** Electrophoresis of 1908_rAAV1-CMV-ZsGreen1 DNA extracted by three methods; 0.5 volume of HS RNA sample buffer was added to a 1-kb DNA ladder (10 ng/μL) by heat treatment (method 1, lane 2); phenol–chloroform extraction/AMPure XP purification (method 2, lane 3); and heat treatment/AMPure XP purification (method 3, lane 4). **(C)** Quantitation of 1908_rAAV1-CMV-ZsGreen1 DNA by ddPCR and TapeStation with three pretreatment protocols. ddPCR, droplet digital PCR; PBS, phosphate-buffered saline; SD, standard deviation.

Next, we examined whether the simple DNA extraction protocol was valid for the TapeStation assay. It has been reported that heat treatment of rAAV particles could release the encapsidated rAAV genome from the packaged capsids.^39^ For extraction of the rAAV genome, vector particles were heated at 95°C for 10 min with or without purification using AMPure XP beads. The extracted rAAV genomes were then analyzed using the TapeStation system (Fig. 5A, B, lanes 1 and 3). For comparison, a sample prepared by the phenol–chloroform extraction/AMPure XP bead purification method was analyzed using the TapeStation system (Fig. 5B, lane 2). These three samples revealed similar yields of genomic DNA quantified using the TapeStation system, which were consistent with the results obtained by ddPCR (Fig. 5C). These results indicated that heat treatment at 95°C for 10 min in PBS is a simple and useful pretreatment of rAAV genome extraction and suitable for TapeStation analysis.

**Figure 6.**
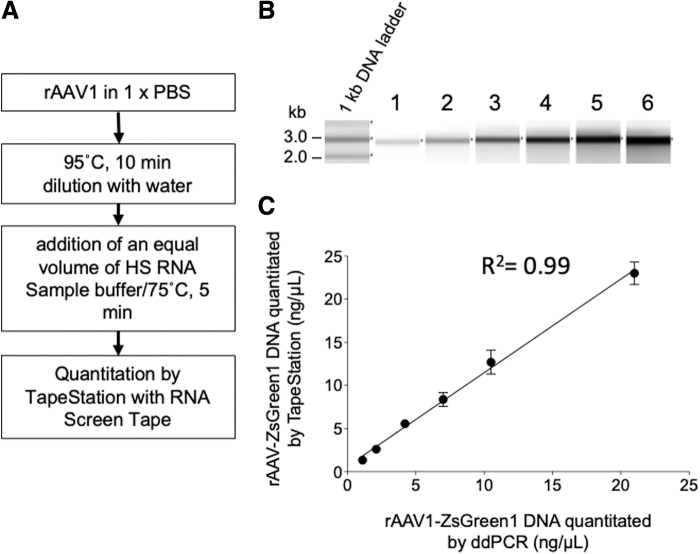
Quantification of packaged rAAV after heat treatment. **(A)** Procedure for sample prep. **(B)** An equal volume of HS RNA sample buffer was added to the 1-kb DNA ladder (10 ng/μL), and the mixture was loaded after heat treatment at 72°C for 5 min (lane 1). An equal volume of HS RNA sample buffer was added to 1–20 ng/μL 1908_rAAV1-CMV-ZsGreen1 (lanes 2–6). **(C)** 1908_rAAV1-CMV-ZsGreen1 concentration quantitated by TapeStation using RNA screen strip was plotted against those quantified by ddPCR.

Serially diluted rAAV1-CMV-ZsGreen1 was analyzed to quantify the rAAV genome using the TapeStation system (Fig. 6A). The amount of rAAV1-CMV-ZsGreen1 DNA quantified using the TapeStation system was plotted against results obtained using ddPCR (Fig. 6B, C). Quantification was available in the range of 1 to 20 ng/μL with a linear regression (*R*^2^ = 0.99), but TapeStation measurement slightly overestimated the values (slope 1.1) compared with ddPCR. These results indicated that PBS did not affect the quantification of ssDNA using TapeStation. However, as per the manufacturer's instructions, attention should be paid to the buffer composition when RNA samples are analyzed using the TapeStation system.

## DISCUSSION

In this study, we demonstrated the feasibility of using an automated electrophoresis device to determine the size, concentration, and integrity of ssDNA extracted from rAAV capsids. One critical quality attribute is the integrity of rAAV ssDNA packaged in the capsids, and it requires appropriate analytical techniques for quality control. The analysis of the size of single-stranded rAAV DNA packaged in capsids used to be performed using alkaline agarose gel electrophoresis.^33^ Current analytical methods for rAAV genome integrity include high-throughput sequencing,^40–42^ two-dimensional digital PCR,^43^ AUC,^22–24^ and capillary gel electrophoresis.^40,44^ However, each of them lacks simplicity, speed, and user-friendliness. In this work, we demonstrate a concise and rapid analysis to characterize the integrity of rAAV genomes. The TapeStation system and its reagents for RNA analysis were used for the rAAV ssDNA analysis. In the electrophoretic analysis, formamide, formaldehyde, and glyoxal are commonly used for RNA.^45–47^ Formamide is ineffective against rigid secondary structures, whereas glyoxal exhibits a relatively strong denaturation effect on secondary structures. Although the specific denaturation reagents used in the system were not disclosed by the manufacturer, the denaturation conditions described in this report addressed concerns regarding the analysis of ssDNA with GC-rich secondary structures such as ITRs. The method proposed in this report offers a rapid and high-throughput alternative for confirming the purity and integrity of the rAAV genome, without the need for training in the use of specialized equipment.

In this system, SYBR Gold was used for detection of ssDNA. SYBR Gold stain is a proprietary, unsymmetrical cyanine dye that exhibits >1,000-fold fluorescence enhancement upon binding to nucleic acids and has a high quantum yield upon binding to ssDNA or to RNA. SYBR Gold stain is more sensitive than ethidium bromide, SYBR Green I stain, and SYBR Green II stain for detecting ssDNA and RNA.^48^ In the TapeStation assay, ssDNA revealed a 1.18-fold higher mobility than that of RNA, and the fluorescence intensity of ssDNA was proportionally increased to the amount of applied ssDNA (*R*^2^ > 0.99) (data not shown).

The TapeStation system is frequently installed in laboratories and supports massive parallel sequencing for quality control of sequencing samples. Depending on the number of samples analyzed, analysis of 15 or fewer samples can be completed within 1 h, including the denaturation treatment. For the analysis, 1–25 ng (7 × 10^9^–1.8 × 10^10^ vg) of rAAV was sufficient to evaluate the ssDNA amount (Fig. 6C) when the rAAV genome size was 2.6 kb, although the concentration range was not broad compared with qPCR.

## CONCLUSIONS

The use of an automated electrophoresis system, the TapeStation, along with appropriate denaturation conditions, enables efficient and accurate evaluation of rAAV ssDNA, providing valuable insights into the purity and integrity of the rAAV genome. This method will represent a valuable contribution to the field, streamlining the rAAV analysis process and enhancing the accessibility of quality control.
